# Masking ability of gingiva-colored resin-based composites over different tooth-colored substrates

**DOI:** 10.1007/s10266-024-00966-6

**Published:** 2024-07-11

**Authors:** Nazmiye Şen, Erkan Sancaklı

**Affiliations:** Faculty of Dentistry, Department of Prosthodontics, School of Dentistry, University of Istanbul, Çapa/Fatih, Istanbul, Turkey

**Keywords:** Masking ability, Gingiva-colored resin-based composite, Color perception, CIELab, CIEDE2000

## Abstract

The purpose of the study was to investigate the influence of different tooth-colored substrates and restoration thicknesses on the final color of gingiva-colored resin-based composites (GCRBCs). Five different shades of GCRBCs [light pink (LP), dark pink (DP), orange (Or), brown (Br), and purple (P)] were used to prepare disc-shaped specimens with 2 different thicknesses: 1.0 mm, and 2.0 mm. GCRBC discs (*n* = 5) were placed over 3 different tooth-colored substrates (ND1, ND5, and ND9) and color parameters were assessed using a spectroradiometer. Color differences (∆E*_ab_ and ∆*E*_00_) were calculated using CIELab and CIEDE2000 formulas and compared to 50:50% perceptibility (PT: ∆E*_ab_ = 1.7, Δ*E*_00_ = 1.1) and acceptability (AT: ∆E*_ab_ = 3.7, Δ*E*_00_ = 2.8) visual thresholds. Color variation data were statistically analyzed using two-way ANOVAs followed by Bonferroni’s post hoc tests (*a* = 05). The ∆E*_ab_ and Δ*E*_00_ values of GCRBCs placed over ND9 substrates were significantly higher in the LP-1.0 mm and Or-1.0 mm groups that presented values above AT (*p*< .001).

Regardless of the substrate color and GCRBC thickness applied, ∆E*_ab_ and Δ*E*_00_ values below AT were recorded in the gingival color groups of P. Substrate color significantly affected the color differences in the gingival color groups of LP, DP, and Or with a restoration thickness of 1.0 mm (*p* < .05). Gingival color, restoration thickness, and substrate color influenced the color differences of GCRBCs. When the gingival color was a lighter gingival color, rather than dark purple, the masking ability was decreased, especially with a restoration thickness of 1.0 mm.

## Introduction

The apical migration of gingival tissues is a highly prevalent pathology that could be treated with restorative, surgical, periodontal, orthodontic, or multidisciplinary treatment approaches [1–4]. Although surgical intervention to reproduce the gingival architecture is the therapeutic approach, gingiva-colored resin-based composites (GCRBC) have been suggested for patients when surgery is not possible or contraindicated [4, 5]. GCRBC has been a conservative alternative that could be used to camouflage the effects of gingival recession [5, 6]. Masking ability, color match and appearance are determining factors to achieve an optimal esthetic outcome with GCRBCs [7–9]. However, masking discolored substrates could be challenging due to several clinical factors, especially in the case of discolored substrates and limited restoration thicknesses [8, 9].

Although gingival color has been considered to be effective on the esthetic outcome, research in this area mainly focused on teeth and tooth-colored restorative materials leaving the importance of gingival color non-emphasized [4, 10]. Human gingiva has been described as highly variable in color, ranging from a pale pink color to a bluish-purple based on visual examination [4, 5]. The color of human gingiva is determined by factors that include epithelium thickness, physiologic melanin pigmentation, and the degree of vascularization which can also be altered by factors such as smoking, systemic disease, age, and medication [4–6]. Healthy gingiva has been classified with representative colors, such as pale pink, dark pink, coral pink, brown, and bluish purple [4, 5]. It has been stated that 80% of the population shows part of their gingival tissues when smiling which emphasizes the importance of gingival color for anterior esthetics and successful restorations [3–5]. Inadequacies in patient satisfaction were observed when focused only on dental (white) esthetics and the interaction between various factors on color perception has not been considered [6, 8, 10]. Consequently, insufficiency to reveal the interaction of various factors including substrate color and restoration thickness on the final color of GCRBCs will result in compromised esthetics [8].

Visual thresholds for color discrimination of dental materials have been used for interpretation of color-related findings and as a quality control tool for standardization of dental shade conformity [11, 12]. According to the international organization for standardization (ISO/TR 28642:2016), color differences should be assessed based on 50:50% perceptibility threshold (PT) and 50:50% acceptability threshold (AT). The color difference between PT and AT is reported to be an acceptable match while the color difference above AT is considered an unacceptable match [2, 13]. CIELab color difference coordinates are traditionally used to evaluate color differences of dental materials [14, 15]. However, the CIELab color space is reported to be nonuniform and different weighting functions (ΔL*, ΔC*, and ΔH^*^) have been introduced with the CIEDE2000 formula to accurately reflect the color difference observed [15].

Masking ability is critical for managing GCRBCs effectively to meet the increasing esthetic expectations of patients for a pleasant smile. However, little emphasis is given to investigate the influence of different gingival colors, restoration thicknesses and substrate colors on the final color GCRBCs. The lack of scientific information on this interaction prevents estimating the effects of various gingiva and substrate colors on the final color of GCRBCs. Therefore, the purpose of this in vitro study was to evaluate the influence of different restoration thicknesses, gingiva, and substrate colors on the final color of GCRBCs by assessing perceptibility and acceptability thresholds. The null hypothesis was that the final color of GCRBCs would not be affected by the tested parameters.

## Material and methods

### Preparation of samples

Five different shades of GCRBCs [light pink (LP), dark pink (DP), orange (Or), brown (Br), and purple (P)] were used to prepare disc-shaped specimens with 2 different thicknesses: 1.0 and 2.0 mm. GCRBC discs (*n* = 5) were placed over 3 different tooth-colored substrates (ND1, ND5, and ND9) to investigate color parameters. The sample size was determined by power analysis, indicating that *n* = 5 in each subgroup provided a power of 95% at a significance level of 0.05.

**Fig. 1:**
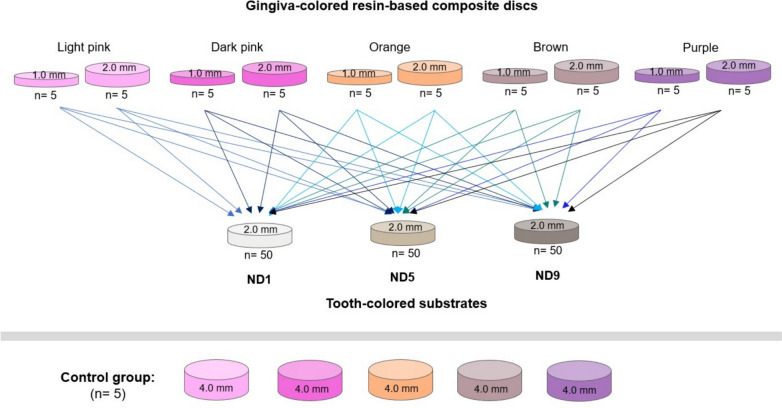
Schematic design of layered specimens

CAD software (Ceramill Mind; Amann Girrbach AG, Koblach, Austria) was used to design transparent indexes (PMMA dental material Ceramill A-Splint; Amann Girrbach AG, Koblach, Austria) with an inner diameter of 12.0 mm, and 3 different inner depths: 1.0, 2.0, and 4.0 mm. Five discs were prepared from each shade of GCRBC in 2 different thicknesses: 1.0, and 2.0 mm. Additionally, one disc-shaped specimen with a thickness of 4.0 mm was prepared for the control group (Fig. 1). GCRBC (Beautifil II Gingiva; Shofu Inc. Kyoto, Japan) was packed into the transparent index and pressed with a glass slab to reach the final shape. Then, light curing was applied from each surface (Bluephase 20i, 1200 mW/cm^2^; Ivoclar Vivadent AG, Schaan, Liechtenstein) for 40 s. The transparent index with an inner depth of 2.0 mm was used for the preparation of tooth-colored substrates (ND1: imitates natural tooth color, ND5: heavily discolored tooth and ND9: extremely discolored tooth/ metallic structures). A total of 150 tooth-colored substrates were prepared using light-activated resin-based composite material (IPS Natural Die Material; Ivoclar Vivadent AG, Schaan, Liechtenstein). The material was packed into the transparent index and covered by a glass slab and light cured for 40 s from the top and bottom surfaces. All specimens were stored at 37 °C distilled water for 24 h before performing color measurements.

### Color measurements

Color parameters were assessed using a non-contact spectroradiometer (PR-670 SpectraScan; Photo Research, Chatsworth, CA, USA) with an illuminating/measuring geometry of 45°/0°. The spectral reflectance values were measured in the wavelength range of 380–780 nm at 2 nm intervals at the center of each specimen with a focus measuring diameter of 1.0 mm. Saturated sucrose solution with a refraction index of 1.5 was placed between GCRBC and substrate [6]. The specimens were placed 40 cm away from the spectroradiometer and three measurements without replacement were performed. The averaged values of spectral reflectance were converted to CIEL*a*b* color coordinates using the CIE D65 Standard Illuminant and CIE 2-degree Standard Observer. Color differences were calculated using CIELab (∆E*_ab_) and CIEDE2000 (Δ*E*_00_) color difference metric according to the following Equations [15, 16]:

$$\Delta E *_{ab} = \left[ {\left( {\Delta L * } \right)^{2} + \left( {\Delta a * } \right)^{2} + \left( {\Delta b * } \right)^{2} } \right]^{{{\raise0.7ex\hbox{$1$} \!\mathord{\left/ {\vphantom {1 2}}\right.\kern-0pt} \!\lower0.7ex\hbox{$2$}}}}$$

∆L*, ∆a*, and ∆b*are the differences in lightness, green–red and blue-yellow color coordinates, respectively.

$$\Delta E00=\sqrt{{\left(\frac{\Delta {L}{\prime}}{{K}_{L}{S}_{L}}\right)}^{2}+{\left(\frac{\Delta {C}{\prime}}{{K}_{C}{S}_{C}}\right)}^{2}+ {\left(\frac{\Delta {H}{\prime}}{{K}_{H}{S}_{H}}\right)}^{2}+{R}_{T}\left(\frac{\Delta {C}{\prime}}{{K}_{C}{S}_{C}}\right)\left(\frac{\Delta {H}{\prime}}{{K}_{H}{S}_{H}}\right)}$$

Δ ${L}{\prime}$, Δ ${C}{\prime}$, and Δ ${H}{\prime}$ correspond to differences in lightness, chroma and hue. RT is the rotation function that accounts for the interaction between chroma and hue differences in the blue region. *K*_L_, *K*_C_, and *K*_H_ are the parametric factors that are set at 1.0 for the present study. Color differences were assessed based on 50:50% perceptibility (PT) and acceptability (AT) visual thresholds described in a reference study to interpret the results [2]. It was considered an excellent match when △E*_ab_ ≤ 1.7 and △*E*_00_ ≤ 1.1. The AT visual thresholds were 1.7 < △E*_ab_ ≤ 3.7 and 1.1 < △*E*_00_ ≤ 2.8 for acceptable match. It was considered moderately unacceptable when 3.7 < △E*_ab_ ≤ 7.4 and 2.8 < △*E*_00_ ≤ 5.6, and clearly unacceptable when 7.4 < △E*_ab_ ≤ 11.1 and 5.6 < △*E*_00_ ≤ 8.4 [2].

***Statistical analysis***

Color variation data were analyzed using a statistical software program (IBM SPSS Statistics, v24; IBM Corp, Armonk, NY, USA). Shapiro–Wilk and Levene tests were used to assess the normality of data distribution. Two-way analyses of variance (ANOVA) followed by Bonferroni comparison tests were applied to analyze color differences (*α* = 0.05).

## Results

**Fig. 2:**
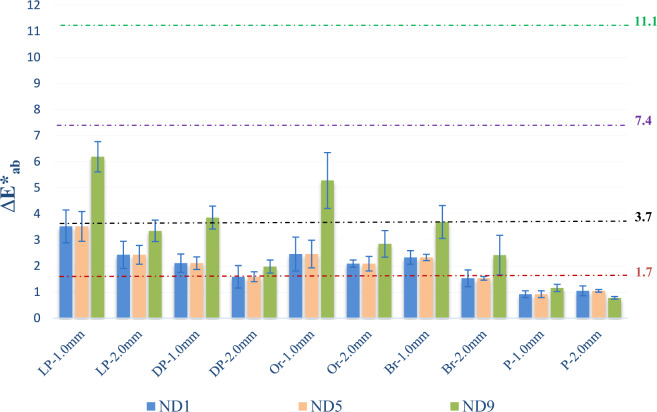
CIELab (∆E*_ab_) color differences of specimens. Horizontal lines represent visual thresholds for 50:50% perceptibility and acceptability: excellent match △E*_ab_ ≤ 1.7; acceptable match 1.7 < △E*_ab_ ≤ 3.7; moderately unacceptable 3.7 < △E*_ab_ ≤ 7.4, and clearly unacceptable 7.4 < △E*_ab_ ≤ 11.1

**Fig. 3:**
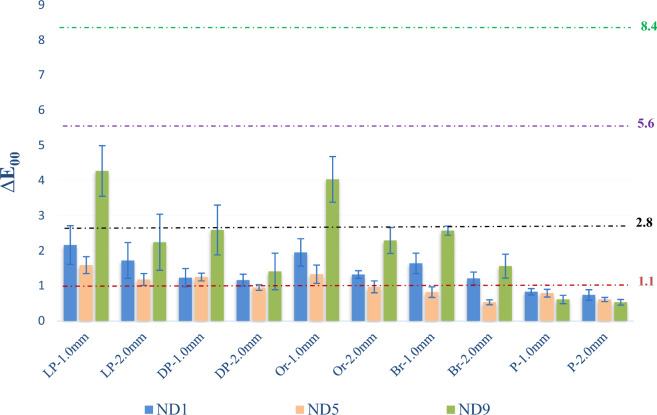
CIEDE2000 (∆*E*_00_) color differences of specimens. Horizontal lines represent visual thresholds for 50:50% perceptibility and acceptability: excellent match △*E*_00_ ≤ 1.1; acceptable match 1.1 < △*E*_00_ ≤ 2,8; moderately unacceptable 2,8 < △*E*_00_ ≤ 5.6, and clearly unacceptable 5,6 < △*E*_00_ ≤ 8,4

CIELab color coordinates of GCRBC discs in the control group are shown in Table 1. The results of color differences among GCRBCs (LP, DP, Or, Br, and P) with different thicknesses and substrate colors are presented in Tables 2 and 3. The mean ∆E*_ab_ and Δ*E*_00_ values of GCRBCs placed over ND9 substrate were significantly higher in the LP-1.0 mm and Or-1.0 mm groups that presented values above AT and were considered as moderately unacceptable (Figs. 2, 3).

**Table 1:** CIELAB color coordinates of specimens in the control group

Gingiva color	L^*^	a^*^	b^*^
Light pink	60.47	21.93	12.80
Dark pink	51.33	29.70	15.37
Orange	55.10	21.17	23.87
Brown	51.25	20.53	14.93
Purple	42.03	12.57	6.20

**Table 2:** Mean and standard deviation values of ∆E*_ab_ considering different gingival colors, thicknesses, and substrate colors

Gingival color	Thickness (mm)	Substrate color			*p
		ND1	ND5	ND9	
Light pink	1.0	3.52 ± 0.63^Ab^	2.14 ± 0.57^Ac^	6.19 ± 0.58^Aa^	< .001
	2.0	2.43 ± 0.52^Ba^	1.93 ± 0.36^Ab^	3.35 ± 0.41^Ba^	< .001
Dark pink	1.0	2.11 ± 0.35^Bb^	1.85 ± 0.24^Ab^	3.86 ± 0.44^Ba^	< .001
	2.0	1.59 ± 0.43^Ba^	1.44 ± 0.19^Aa^	1.98 ± 0.25^Ca^	.302
Orange	1.0	2.46 ± 0.65^Bb^	2.21 ± 0.53^Ac^	5.28 ± 1.07^Aa^	< .001
	2.0	2.09 ± 0.14^Ba^	1.65 ± 0.28^Aa^	2.85 ± 0. 51^Ba^	.075
Brown	1.0	2.33 ± 0.26^Bb^	1.33 ± 0.12^Ab^	3.69 ± 0.63^Ba^	< .001
	2.0	1.53 ± 0.32^Ba^	0.81 ± 0.07^Bb^	2.42 ± 0.76^Ba^	< .001
Purple	1.0	0.92 ± 0.13^Ba^	1.04 ± 0.13^Ba^	1.16 ± 0.14^Ca^	.216
	2.0	1.05 ± 0.19^Ba^	0.96 ± 0.05^Ba^	0.78 ± 0.05^Ca^	.384
**p		.014	.003	< .001	

**Different uppercase letters indicate the presence of significant difference between different gingival colors in the same substrate color group (column). *Different lowercase letters indicate the presence of significant differences between substrate colors in the same gingival color group (row)

**Table 3:** Mean and standard deviation values of ∆*E*_00_ considering different gingival colors, thicknesses, and substrate colors

Gingival color	Thickness (mm)	Substrate color			*p
		ND1	ND5	ND9	
Light pink	1.0	2.16 ± 0.55^Ab^	1.59 ± 0.24^Ac^	4.27 ± 0.72^Aa^	< .001
	2.0	1.72 ± 0.51^Aa^	1.18 ± 0.17^Aa^	2.24 ± 0.80^Ba^	.056
Dark pink	1.0	1.23 ± 0.26^Ab^	1.25 ± 0.11^Ab^	2.59 ± 0.71^Ba^	< .001
	2.0	1.16 ± 0.17^Aa^	0.95 ± 0.08^Aa^	1.41 ± 0.52^Ca^	.168
Orange	1.0	1.95 ± 0.39^Ab^	1.33 ± 0.26^Ac^	4.03 ± 0.65^Aa^	< .001
	2.0	1.32 ± 0.11^Aa^	0.97 ± 0.17^Ab^	2.29 ± 0.37^Ba^	< .001
Brown	1.0	1.64 ± 0.29^Aa^	0.82 ± 0.15^Ab^	2.57 ± 0.13^Ba^	< .001
	2.0	1.21 ± 0.18^Aa^	0.53 ± 0.07^Ab^	1.56 ± 0.34^Ca^	< .001
Purple	1.0	0.83 ± 0.09^Ba^	0.79 ± 0.11^Aa^	0.61 ± 0.12^Da^	.343
	2.0	0.74 ± 0.15^Ba^	0.51 ± 0.06^Aa^	0.53 ± 0.08^Da^	.251
**p		.017	.080	< .001	

**Different uppercase letters indicate the presence of significant difference between different gingival colors in the same substrate color group (column). *Different lowercase letters indicate the presence of significant differences between substrate colors in the same gingival color group (row)

Substrate color significantly influenced the ∆*E* values of GCRBCs (*p* < 0.001) (Tables 2, 3). Significantly higher mean ∆*E* values were registered for the substrate color of ND9 in the gingival color groups of LP-1.0 mm, DP-1.0 mm, and Or-1.0 mm (Tables 2, 3).

Regardless of the substrate color and GCRBC thickness applied, ∆E*_ab_ and Δ*E*_00_ values below AT were found for the gingival color groups of P (Figs. 2, 3).

GCRBC thickness significantly influenced ∆E*_ab_ and Δ*E*_00_ values in the gingival color groups of LP, DP, and Or that were placed over ND9 substrates (*p* < 0.05). GCRBC thickness did not influence the mean Δ*E*_00_ values in the substrate color groups of ND1, and ND5 for the gingival colors of LP, DP, Or, Br, and P (*p* > 0.05) (Table 3).

Figures 2 and 3 represent the color differences of GCRBCs with different thicknesses and substrate colors according to 50:50% AT (∆E*_ab_ = 3.7, Δ*E*_00_ = 2.8) and 50:50% PT (∆E*_ab_ = 1.7, Δ*E*_00_ = 1.1) visual thresholds. Color measurements obtained in the groups of LP-1.0 mm and Or-1.0 mm presented color differences above AT (Figs. 2, 3).

## Discussion

The present study was designed to investigate the influence of different gingival colors, restoration thicknesses, and substrate colors on the masking ability of GCRBCs. The null hypothesis was rejected based on the results revealing significant interactions of gingival color, restoration thickness, and substrate color on both ∆E*_ab_ and Δ*E*_00_ values of GCRBCs.

Color and appearance have been shown to be the key factors in determining the success of restorations in esthetic dentistry [8, 16, 17]. However, research related to color has been focused on teeth and related restorative materials leaving the importance of gingival color non-emphasized [8, 16, 17]. Several studies have been conducted to find out the complex balance of factors effective on the final color of restorations [1, 7, 18]. Clinically relevant testing of color perception can help to better understand the complex nature of color and factors effective on the final color of GCRBCs [7, 8]. In the present study, five different gingival colors with 2 different thicknesses were placed over 3 different tooth-colored substrates. For this purpose, tooth-colored composite material designed for simulating different tooth shades was used. The use of tooth-colored substrates as backgrounds could provide a clinically reliable approach for evaluating color differences rather than black and white backgrounds as suggested by the International Organization for Standardization, ISO/TR 28642–2016 [7, 13].

The color of GCRBCs could be visually or instrumentally assessed using spectrophotometers or spectroradiometers [1, 2, 6, 7]. In the present study, a spectroradiometer was used for color measurements. Spectroradiometers provide noncontact color measurements and unlike spectrophotometers do not contain a stable light source [2]. In addition, color measurements with spectroradiometers can be achieved under the same viewing conditions as human observers [17, 19]. Different color systems and formulas are used to assess the color of restorations recognized by the human eye [10–12]. Traditionally, the CIELab color difference formula is used for the assessment of perceptibility and acceptability of color differences [17]. However, CIELab color space is reported to be non-uniform and CIEDE2000 color difference formula is recommended to achieve a better correlation with visual perception [10–12]. In the present study, the results of color differences were presented using both CIELab and CIEDE2000 color difference formulas. For the interpretation of color differences, PT and AT visual thresholds were used to correlate the numerical data with what is perceived and visualized clinically [2].

In the present study, ∆E*_ab_ and Δ*E*_00_ values of GCRBCs were significantly affected by different gingival colors, thicknesses, and substrate colors. Significantly higher ∆E*_ab_ and Δ*E*_00_ values were found in the LP-1.0 mm and Or-1.0 mm groups layered over ND9 substrates that presented values considered as moderately unacceptable. GCRBC thickness influenced ∆*E*_00_ values in the gingival color groups of LP, DP, Or, and Br when layered over ND9 substrates. Higher color differences were found in the GCRBC groups (LP, DP, Or, and Br) with a thickness of 1.0 mm when compared to the thickness of 2.0 mm. A recent study by Pérez et al. compared the influence of different background colors and thicknesses on the appearance of gingiva-colored composite resins. In accordance with the present study, they reported significantly higher color differences for the restorations with a thickness of 1.0 mm [8]. In the present study, color measurements obtained over ND9 substrates showed higher ∆E*_ab_ and Δ*E*_00_ values than the measurements obtained over ND1 and ND5 substrates for the gingival color groups of LP, DP, Or, and Br. More specifically, changing the gingival color from a lighter pink to a darker purple color resulted in decreased ∆E*_ab_ and Δ*E*_00_ values. Furthermore, thickness and substrate color did not significantly influence the color differences in the gingival color groups of P. Recently, Gouveia et al. investigated the influence of different background colors and thicknesses on the final color of gingiva-colored composites. In line with the present results, the authors concluded that background color and gingiva shade influenced the final color of gingiva-colored composites [9]. In addition to the studies on GCRBCs, several studies evaluating visual thresholds of tooth-colored restorative materials have been conducted [7–9]. The research on restorative materials has shown the effect of several factors on color perception, such as environmental background, illumination source, substrate color, restoration thickness, cement shade and measurement technique [7–9, 18, 20]. It has been reported that the color of background and restoration thickness influence the color differences of restorative materials [7, 9].

As a limitation of the present study, only one brand of GCRBC material was used. Additionally, color measurements were performed using only instrumental color measurement device. Further research of masking ability is needed, accompanying instrumental color measurements with human visual assessments, which could help to confront the visual thresholds and to have a better perceptive information about the clinical acceptability of GCRBCs. In vivo studies assessing the effect of different material brands, gingiva, and substrate colors would also be useful to verify the blending effect of several parameters on the color perception of GCRBCs.

## Conclusions

Within the limitations of the present study, the following conclusions were drawn:

1. Masking ability of GCRBCs was affected by different gingival colors, thicknesses, and substrate colors.
2. Darker gingival colors showed greater masking ability. When the gingival color was a lighter gingival color, rather than dark purple, the masking ability was decreased especially in the case of 1.0 mm restoration thickness.
3. Thickness should be considered to derive the best clinical outcome concerning the masking ability of GCRBCs over discolored substrates.

## Data Availability

The data that support the findings of this study are available from the corresponding author upon reasonable request.
